# The Ipswich Meeting of the British Medical Association

**Published:** 1900-07-28

**Authors:** 


### The Ipswich Meeting of the British Medical Association.

The meeting of the British Medical Association at Ipswich next week is likely to be full of interest, for, besides the attractive social and scientific programme which has been arranged, it is not unlikely that the discussions at the general meetings will involve questions of importance, and may perchance be tinged with some acerbity. The question which is just now agitating the Association is, Who is to be head? Are its affairs, its policy, as well as the mere details of management, to be controlled by the Council, or by the general meetings. Hitherto the Council has had everything its own way, and it is to be admitted that it has sometimes paid but scant attention to the mandates of the general meetings. Confident in its position and holding fast to the faith in which it has been brought up, the Council has persistently declined to budge an inch, professing to believe, as indeed was in some cases pretty obvious, that the orators and the little cliques that seemed to enjoy themselves so vastly in passing more or less contradictory resolutions at these meetings had no substantial following. Thus the little feud went on until Council-baiting, and in old days editor-baiting, were coming to be regarded as among the stock amusements provided for the delectation of the members. With a cynical indifference to the elocutionary rights of man, which is still treasured up against it as an example of its deep treachery, the Council stole away the the audience of the reformers by "a lot of confounded garden parties"; but now the tables are turned, for it has at last been discovered that resolutions duly passed at the general meetings, even by a trumpery quorum, are valid and binding. Herein lies a great and growing danger. Ostentation has its penalties, and unfortunately the British Medical Association is not only wealthy, but has boasted of it. The exploitation of its money bags then becomes an object of much attractiveness, and we find that notices of motion have already been sent in for the Ipswich meeting, which if carried will tend to disburden the Association pretty quickly of much of its accumulated treasure. One proposal is that the 21 members of the Journal and Finance Committee shall meet every month, receiving five guineas apiece per day, together with first-class railway fare, that the other members of the Council shall have similar although smaller pay, also with their railway fares, and that the President of the Council shall receive an honorarium of £300 a year; another proposes the creation of a separate medico-ethical and medico-political department, with paid secretary and annual conference, the committee by whom this department is to be organised being also paid at the same rate as the members of Council; another proposal is "that the Council of the British Medical Association set apart a sum of £10,000 towards the organisation and improving the status of the medical profession;" another that paid secretaries be provided for each of the branches, the salary being "not less than £100 per annum with expenses;" and finally another, which, among other things, proposes that a sum of not more than £2,000 a year out of the funds of the Association shall be placed in the hands of a committee to be appointed for the purpose of carrying into effect "a system of individual and collective medical defence." These are samples of the sort of things that are being seriously proposed, and that might be carried by a small clique at any general meeting, and thus become binding were the general meetings to be "paramount," as indeed it is now held that they are. It is evident that no association could retain any trace of stability if, by the snatch vote of any small group of members, its resources could be so frittered away. Herein, then, lies much of the interest of the Ipswich meeting. That the Council is fully cognisant of the danger may be judged of by the extreme character of the proposals made at the meeting held last week at Exeter Hall, which, if they had happened to be carried, would have most effectually clipped the wings of the outside and unattached members. But they were not carried; in fact, they were somewhat ignominiously rejected. The Council played a game of bluff, and not only lost, but irritated its enemies, and unless a considerable number of the members who go to Ipswich are prepared to face the heat, the noise, the weariness, and the endless flow of words which generally characterise the general meetings, and to vote solid for the Council, it is quite on the cards that some preposterous resolution may be carried which will bring the affairs of the Association to a very unpleasant climax.

That some reform is necessary no one can doubt, for even if we grant that all power ought to be derived from the branches and be centred in the Council, still some form of Referendum might well be instituted by aid of which the collective opinion of the branches on any special question might be obtained, and might have binding force. That would be a reasonable way of tempering the autocratic power of the Council. But to hand over the control of the policy and funds of the Association to the general meetings, and to such "common sense" as might happen to be found there, would mean ruin and destruction.

